# Drought Sensitivity of Sugarcane Cultivars Shapes Rhizosphere Bacterial Community Patterns in Response to Water Stress

**DOI:** 10.3389/fmicb.2021.732989

**Published:** 2021-10-21

**Authors:** Qi Liu, Sasa Xie, Xiaowen Zhao, Yue Liu, Yuanjun Xing, Jicao Dao, Beilei Wei, Yunchang Peng, Weixing Duan, Ziting Wang

**Affiliations:** ^1^Guangxi Key Laboratory of Sugarcane Biology, Nanning, China; ^2^State Key Laboratory for Conservation & Utilization of Subtropical Agro-Bioresources, Guangxi University, Nanning, China; ^3^College of Agronomy, Guangxi University, Nanning, China; ^4^Sugarcane Research Institute, Guangxi Academy of Agricultural Sciences, Nanning, China

**Keywords:** drought stress, sugarcane, rhizosphere bacterial community, environmental factor, drought response

## Abstract

Rhizosphere bacteria, the main functional microorganisms inhabiting the roots of terrestrial plants, play important roles in regulating plant growth and environmental stress resistance. However, limited information is available regarding changes occurring within the structure of the root microbial community and the response mechanisms of host plants that improve adaptability to drought stress. In this study, we conducted an experiment on two sugarcane varieties with different drought tolerance levels under drought and control treatments and analyzed the rhizosphere bacterial communities using 16S rRNA high-throughput sequencing. Correlation analysis results clarified the influence of various factors on the rhizosphere bacterial community structure. Drought stress reduced the diversity of the bacterial community in the rhizosphere of sugarcane. Interestingly, the bacterial community of the drought-sensitive sugarcane cultivar GT39 changed more than that of the drought-tolerant cultivar ZZ9. In addition, ZZ9 had a high abundance of drought-resistant bacteria in the rhizosphere under optimal soil water conditions, whereas GT39 accumulated a large number of drought-resistant bacteria only under drought stress. GT39 mainly relied on Actinobacteria in its response to drought stress, and the abundance of this phylum was positively correlated with soil acid phosphatase and protease levels. In contrast, ZZ9 mainly relied on Bacilli in its response to drought stress, and the abundance of this class was positively correlated with only soil acid phosphatase levels. In conclusion, drought stress can significantly reduce the bacterial diversity and increase the abundance of drought-resistant bacteria in the sugarcane rhizosphere. The high abundance of drought-resistant bacteria in the rhizosphere of drought-tolerant cultivars under non-drought conditions is an important factor contributing to the high drought adaptability of these cultivars. Moreover, the core drought-resistant bacteria of the sugarcane rhizosphere and root exudates jointly affect the resistance of sugarcane to drought.

## Introduction

Plants grow in dynamic environments to which they exhibit remarkable adaptations (Butt et al., 2019; Kundariya et al., 2020). However, adverse environmental changes can cause abiotic stresses, which not only affect the normal growth and development of plants but also threaten their survival (Gupta and Huang, 2014). Drought stress is one of the most common environmental stresses and is capable of reducing major crop yields by 50%–80% (Buchanan et al., 2015; Griffiths and Paul, 2017). In response to environmental stress, plants can form symbiotic relationships with microorganisms. Soil microorganisms are reportedly more sensitive than plants to changes in the soil environment (Waldrop and Firestone, 2006; Lee et al., 2019). Plant roots interact with many soil microbes to form a unique rhizosphere microbial community that participates in the responses to environmental conditions and allows plants to achieve optimal growth and development (Jordan et al., 2016; Meijuan et al., 2018). Although the mechanisms associated with plant responses to drought stress have been extensively studied in terms of morphology, physiology, and genetics, the effects of drought on soil microorganisms remain poorly understood (Zhao et al., 2016; Aimin et al., 2018; Li P. et al., 2019). Investigation of these effects could provide insights into the resistance of plants to environmental stress (Jurburg et al., 2017; Krause et al., 2018).

Plants employ various strategies to overcome drought stress, including a combination of stress avoidance and regulation of drought tolerance, depending on their genotypes (Chaves et al., 2002; Verma et al., 2020). For instance, plants can better resist drought by regulating stomatal closure and expressing drought-related genes (Cao et al., 2017; Koech et al., 2019). Plant roots, owing to their roles in nutrient and water absorption, are important organs for sensing and responding to soil water stress (Iquebal et al., 2019; Teramoto et al., 2020). The interaction between plants and the soil microorganisms that colonize the rhizosphere and root system is considered a key factor in the rapid adaptation of plants to soil environmental stress (Giehl and von Wirén, 2014; Pascale et al., 2020). Plant growth-promoting bacteria have been shown to enhance the water absorption capacity of plant roots under water shortage conditions (Sade et al., 2010; Khan et al., 2020). Specifically, drought stress can induce the growth of certain gram-negative bacteria and increase osmotic pressure, maintain turgor pressure, and protect the macromolecular structures of plant tissues (Welsh, 2000; Schimel et al., 2007). Yasmin et al., found that plant growth-promoting bacteria could reduce malondialdehyde content in plants under drought stress, promote the synthesis of proline and related hormones, and significantly improve plant drought resistance (Yasmin et al., 2020, 2021). However, the changes in the rhizosphere microbial community that occur under drought stress depend on the effects of the host plants and surrounding soil (Naylor and Coleman-Derr, 2018). Differences in plant varieties and genotypes lead to varying levels of drought resistance. For instance, drought-resistant plants have evolved different strategies for adapting to drought compared with water-sensitive plants (Hilker et al., 2016; Yao et al., 2018), and they can rapidly activate drought resistance mechanisms at the physiological and molecular levels in response to drought stress (Zhao et al., 2016; Yao et al., 2018). In addition, the health of host plants and the nutrient conditions in the soil surrounding the roots can alter the microbial composition of the rhizosphere by influencing root structure and exudates (Benitez et al., 2017; Wen et al., 2020). Further, the availability of soil nutrients, such as nitrogen, regulates the diversity of rhizosphere microorganisms (Howard et al., 1995; Treseder, 2008; Kavamura et al., 2013). However, quantifying the effects of various factors on rhizosphere microbes under drought stress is complex, and this requires further exploration.

Sugarcane is an important crop for bioenergy production. It contributes toward 80% of the raw materials used in the global sugar industry, and it is cultivated in more than 110 countries (Garsmeur et al., 2018; Hajar-Azhari et al., 2018). Sugarcane is highly sensitive to water deficit during vegetative growth. Specifically, extreme water shortage reduces the accumulation of sugarcane stalk biomass by up to 60% (Gentile et al., 2015; Ghannoum, 2016). Most sugarcane studies have focused on improving sugar yield and breeding abiotic stress-resistant cultivars (Siraree et al., 2017). However, a large knowledge gap remains regarding the stress resistance of sugarcane rhizosphere microbes, which has been explored in other plants (Zhao et al., 2020b). For example, the rhizosphere microorganisms of different core communities constructed from unique varieties of tomato, citrus, and chickpea plants functioned to improve their environmental adaptability (Demers et al., 2015; Navid et al., 2015; Jin et al., 2018). Similarly, genetically diverse rice species responded to drought stress by utilizing specific rhizosphere microbial communities (Andreo-Jimenez et al., 2019). We hypothesized that the diversity of the rhizosphere microbial community of different sugarcane varieties resulted from the long-term selection and shaping of the rhizosphere environment by the host sugarcane, which could help it adapt to adversity. Thus, certain sugarcane varieties could respond better to water shortage under drought stress.

The current study, therefore, sought to analyze the major changes in sugarcane rhizosphere microbial diversity under drought stress. We intended to provide insights into not only the general responses of soil microbes to environmental stress but also the sugarcane-specific rhizosphere bacterial community. To this end, we selected ZZ9, a sugarcane cultivar with strong drought resistance, and GT39, a drought-sensitive cultivar, to evaluate their microbial communities under drought and normal water conditions. The primary questions that were addressed in this study were as follows: (1) What changes in the sugarcane rhizosphere bacterial community structure are induced by drought stress? (2) What are the main effects of sugarcane cultivar drought tolerance on the rhizosphere bacterial community? (3) What is the response pathway of drought-resistant bacteria in the sugarcane rhizosphere under drought stress?

## Materials and Methods

### Cultivar Selection and Field Experiment Design

This study was performed in the Sugarcane Variety R&D and Breeding Base, Fusui, China (between 107°310′ and 108°060′ E and 22°170′ and 22°570′ N; 83 m a.s.l.) of Guangxi University. Experiments were carried out in the summer of 2018. The experimental site is located in a subtropical monsoon climate, with long summers and short winters. The average annual temperature at the experimental site is 22°C. In this region, the annual sunshine time is approximately 2600 h, and the annual precipitation is 1050–1300 mm. The soil used in the experiments was collected from the top layer (0–20 cm) of a long-term sugarcane cultivation field with the following physicochemical characteristics: pH, 6.15; organic matter content, 19.47 g/kg; total nitrogen content, 100.5 g/kg; total phosphorus content, 22.4 g/kg; total potassium content, 7.11 g/kg; alkaline hydrolyzed nitrogen, 136 mg/kg; available phosphorus, 83 mg/kg; and available potassium, 77.1 mg/kg. The sugarcane varieties selected were Zhongzhe9 (ZZ9) and Guitang39 (GT39). GT39 is a high-yield species bred through sexual hybridization. Its female parent is Yuetang93/159, and its male parent is ROC22. GT39 is more sensitive to water and fertilizer conditions than its parents (Wang et al., 2013). The female parent of ZZ9 is ROC22, and the male parent is Yunzhe89-7. Under drought conditions, single index analysis and comprehensive membership function evaluation of stomatal characteristics, physiological changes, and molecular mechanisms showed that ZZ9 is more resistant to drought than its parents (Yin et al., 2020). Because the two cultivars exhibit a large difference in sensitivity to water, we used GT39 as a water-sensitive variety and ZZ9 as a drought-resistant (drought-tolerant) variety in these experiments.

The sugarcane was planted in flowerpots and cultivated in a glass greenhouse. The average temperature in the greenhouse was 22°C, consistent with the outside temperature, and the plants were only exposed to natural light. The upper diameter of the plastic pots was 35 cm, the lower diameter was 25 cm, and the height was 35 cm; three drainage holes, with a diameter of approximately 1 cm, were drilled in the bottom of each pot (Supplementary Figure 1). A total of 60 pots were used for planting, 30 for GT39 and 30 for ZZ9, each with an average of two to three sugarcane plants (Supplementary Figure 2). When the plants sprouted 2–3 leaves, 12 pots of each cultivar were selected for follow-up experiments; of these pots, six per cultivar were subjected to drought stress (Drought, D), and the remaining six were watered normally (Control, C). Soil water content was measured at a depth of 10–15 cm using a TDR-100 soil moisture meter (Spectrum Technologies, Inc., Aurora, IL, United States). Irrigation was based on the weekly water requirements of sugarcane, and regular watering was carried out in the early stage of planting and continued for the control plants. For drought treatments, drought stress was induced by stopping irrigation. In a previous study, the chlorophyll content of the sugarcane leaves decreased under soil water deficit, and this was more pronounced in varieties with low drought tolerance (Luo and Liang, 2005; Qin et al., 2017). Hence, in this study, chlorophyll content was used to indicate the level of drought stress.

### Soil Sampling and Assessment of Physical and Chemical Properties

Soil samples were collected from the soil attached to the surface of the sugarcane roots; the soil tightly attached to the roots was regarded as rhizosphere soil. At the end of the drought stress period (day 31), two plants with uniform growth were selected from each group, and the root system was dug out with surrounding soil (surface area of approximately 20 cm^2^ around the plant). Large soil lumps and stones were removed, loose soil was gently shaken off, and the rhizosphere soil attached to the root surface was collected with a brush. The collected soil was sieved through a 2 mm sieve. Three pots for each group were sampled for a total of 12 soil samples collected from the four treatment groups (GT39D, GT39C, ZZ9D, and ZZ9C; D represents drought, and C represents the control). Each soil sample was divided into two sub-samples. One was stored at −40°C and was used to measure the physical and chemical properties of the soil, and the other was stored at −80°C for the extraction of rhizosphere microbial DNA, which was done within 24 h (Zhao et al., 2020a).

The chemical properties of the soil were determined as previously described (Bao, 2000). Soil organic carbon (SOC) was determined by the potassium dichromate sulfuric acid oxidation method, total nitrogen (TN) was measured using the semi-micro Kelvin method, and available phosphorus (AP) was determined by molybdenum antimony colorimetry. Measurements were conducted on soil collected from a depth of approximately 10–15 cm. Leaf chlorophyll content was measured with a chlorophyll meter (SPAD-502 Plus; Spectrum Technologies, Aurora, IL, United States). Three leaves from three plants for each treatment group were selected for chlorophyll content determination. The water potential of the leaves was measured with a dew point water potential meter (WP4; Decagon Devices, Inc., Pullman, WA, United States) between 11:30 and 12:00 using the youngest fully expanded leaves. To determine leaf water potential, we selected five points from the tip to the base of each leaf, and the measurement was repeated on three different plants for each treatment group. The root-shoot ratio was calculated as the root dry weight divided by the stem dry weight. Soil enzyme activity was determined as previously described (Shao and Li, 2016). Soil catalase (S-CAT) activity was measured using a volumetric method, and soil acid phosphatase (S-ACP), soil urease (S-UE), and acid protease (S-ACPT) activities were measured using colorimetry. For biomass determination, each plant was divided into shoots (including all aerial components) and roots, and their biomasses were determined after drying at 80°C.

### DNA Extraction, Bacterial 16S Gene Polymerase Chain Reaction Amplification, and Sequencing

Soil DNA was extracted using the E.Z.N.A. Soil DNA Kit (Omega Bio-Tek, Inc., Norcross, GA, United States) according to the manufacturer’s instructions. Microbial DNA was extracted from a 1 g sample of fresh soil, and the extraction was performed three times for each sample. The concentration and quality of DNA samples were measured with a NanoDrop One spectrophotometer (Thermo Fisher Scientific, Waltham, MA, United States). The primer pair F515 (5′-GTG CCA GCM GCC GCG GTA A-3′) and R806 (5′-GGA CTA CHV GGG TWT CTA AT-3′), targeting the V3–V4 hypervariable regions of the bacterial 16S rRNA gene, was used for polymerase chain reaction (PCR) (Peiffer et al., 2013). The reverse primer contained a 12 bp error-correcting barcode unique to each sample. Primers were synthesized by Invitrogen (Carlsbad, CA, United States). Each reaction was conducted in a volume of 50 μL, containing 25 μL of 2 × Premix Taq (Takara Biotechnology, Dalian Co. Ltd., China), 1 μL of each primer (10 M), and 3 μL of DNA (20 ng/μL) template, using an S1000 thermal cycler (Bio-Rad Laboratories, Inc., Foster City, CA, United States). The following cycling conditions were used: 94°C for 5 min; followed by 30 cycles of denaturation at 94°C for 30 s, annealing at 52°C for 30 s, and extension at 72°C for 30 s; with a final elongation step at 72°C for 10 min. The PCR products were sequenced by Magigene Technology (Guangzhou, China) on the Illumina HiSeq 2500 platform.

Quality filtering of the paired-end raw reads was performed under specific conditions to obtain high-quality, clean reads using Trimmomatic (V0.33^1^) as the quality-control process. Paired-end clean reads were merged using FLASH (V1.2.11^2^) based on the overlap between paired-end reads; spliced sequences with at least 10 overlapping reads generated from the opposite ends of the same DNA fragment and with the maximum allowable error ratio in the overlap region of 0.1 were designated Raw Tags (Caporaso et al., 2010). Sequences were assigned to each sample based on their unique barcode and primer using Mothur (V1.35.1^3^). Then, the barcodes and primers were removed to obtain effective clean tags. Further sequence analysis was performed using Usearch (V10^4^) to filter and eliminate noise from the data by clustering similar sequences with less than 3% dissimilarity, and the Quantitative Insights Into Microbial Ecology (QIIME) pipeline (VirtualBox Version 1.1.0) was used to select 16S rRNA operational taxonomic units (OTUs) from the combined reads of clustered OTUs with 97% similarity (Edgar, 2010). The 16S rRNA gene sequences obtained in this study have been deposited in the National Center for Biotechnology Information Sequence Read Archive database^5^ under accession number PRJNA655948.

### Statistical and Bioinformatics Analyses

The species diversity of each sample was analyzed based on three alpha diversity indices: Chao1, Shannon, and Fisher. All sample indexes were calculated using QIIME (V1.9.1) (Caporaso et al., 2010), and the correlation between the alpha diversity index and environmental factors was analyzed using the corrplot package (Wei et al., 2017) in R (V3.6.3). Beta diversity analysis was used to evaluate differences in terms of species complexity. Weighted and unweighted UniFrac beta diversity indexes were calculated by QIIME. R and local Perl scripts (Perl version 5.6.1) (Tisdall, 2001) were used to generate the sample distance heatmap based on the Bray-Curtis dissimilarity matrix. The relationship between bacterial communities and environmental factors was analyzed using the Mantel test. Heat trees (R package1) (Yu et al., 2017) were used to show the effect of the variables (variety and water stress) on the main flora, and edgeR (Robinson et al., 2010) was used to determine the most differentially abundant bacterial communities between the two treatments. The Mantel test, principal coordinates analysis (PCoA), and distance-based redundancy analysis (dbRDA) were performed using the vegan package in R v3.6.3 (Oksanen et al., 2013).

Molecular ecological network analysis (MENA)^6^ was used to demonstrate the relationship between the rhizosphere bacterial communities of the two sugarcane cultivars and environmental factors (Li Y. et al., 2019). Networks were constructed for the root-associated area and the soil communities based on the relative abundances of OTUs, resulting in two networks. All analyses were performed using the MENA pipeline, and networks were graphed using Cytoscape 2.8.2 (Shannon et al., 2003). We also characterized the modularity of each network created in this study. A module is a group of nodes (OTUs) that are highly connected within the group with few connections outside the group (Newman, 2006).

## Results

### Physical and Chemical Properties of Rhizosphere Soil and the Physiological State of Sugarcane

The SOC, TN, and AP contents in the rhizosphere soil were higher under the control treatment than under drought treatment. Soil organic carbon and TN were significantly correlated with cultivar and water treatment, whereas AP was significantly correlated only with drought stress (Table 1). Soil enzyme activities were significantly correlated with both cultivar and water content. The S-CAT content in the GT39 rhizosphere soil was higher than that in the ZZ9 soil under both the control and drought treatments, whereas the S-ACPT content was higher in the ZZ9 rhizosphere soil than in the GT39 soil. S-UE and SOC showed no significant variability among the four different treatments. Analysis of the physiological indexes of the two cultivars under control and drought treatment revealed that the root-shoot ratio, chlorophyll content, plant biomass, and root biomass under control and drought treatments were higher in ZZ9 than in GT39 (Table 2). In addition, water treatment had a significant effect on all physiological indexes, including leaf water potential, chlorophyll content, and plant biomass; this was significantly correlated with the cultivar. This confirms the high drought resistance of ZZ9 compared with that of GT39.

**TABLE 1:** Physical and chemical properties of soil under different treatments.

Line	Treatment	Soil water content (%)	SOC (g⋅kg^–1^)	TN (g⋅kg^–1^)	AP (g⋅kg^–1^)	S-CAT (U⋅g^–1^)	S-ACP (nmol⋅d^–1^⋅g^–1^)	S-UE (U⋅g^–1^)	S-ACPT (U⋅g^–1^)
GT39	Control	17.55b	8.03b	107.90c	22.63b	21.89d	1.42a	2.17c	1.02a
	Drought	4.98a	5.70a	55.60a	21.40a	15.36c	2.01c	1.64b	1.81b
ZZ9	Control	18.23b	11.27c	94.52b	22.27b	12.86b	1.59b	1.62b	2.12c
	Drought	5.01a	7.24ab	57.35a	21.31a	12.24a	2.77d	1.38a	2.17c
ANOVA	Line	*P* = *0.842*	*P* < *0.001*	*P* < *0.001*	*P* = *0.180*	*P* < *0.001*	*P* < *0.001*	*P* < *0.001*	*P* < *0.001*
	Treatment	*P* < *0.001*	*P* < *0.001*	*P* < *0.001*	*P* < *0.001*	*P* < *0.001*	*P* < *0.001*	*P* < *0.001*	*P* < *0.001*
	Line × Treatment	*P* < *0.468*	*P* = *0.070*	*P* < *0.001*	*P* = *0.407*	*P* < *0.001*	*P* < *0.001*	*P* = *0.003*	*P* < *0.001*

*Values are mean of three soil samples.*

*Different letters indicate significant differences (ANOVA, *P* < 0.05, Tukey’s HSD *post hoc* analysis) among tillage treatments.*

**Soil water content* water content, *SOC* soil organic carbon, *TN* total nitrogen, *AP* available phosphorous, *S-CAT* solid-catalase, *S-ACP* solid-acid phosphatase, *S-UE* soil urease, *S-ACPT* solid -acid protease.*

*Significant at 0.001 level.*

**TABLE 2:** Plant traits of different varieties under different water treatments.

Line	Treatment	Root shoot ratio	Leaf water potential (pd⋅MPa^–1^)	Chlorophyll content (SPAD)	Plant biomass (g)	Root biomass (g)
GT39	Control	0.0999a	−0.276c	28.23c	433.99b	43.36a
	Drought	0.1064ab	−1.286a	11.33a	375.27a	39.92a
ZZ9	Control	0.1200b	−0.185d	29.43c	477.29c	57.28b
	Drought	0.1482c	−0.863b	14.83b	433.28b	64.22b
ANOVA	Line	*P* = *0.001*	*P* < *0.001*	*P* < *0.001*	*P* < *0.001*	*P* = *0.373*
	Treatment	*P* < *0.001*	*P* < *0.001*	*P* < *0.001*	*P* < *0.001*	*P* < *0.001*
	Line × Treatment	*P* = *0.014*	*P* < *0.001*	*P* = *0.032*	*P* = *0.223*	*P* = *0.023*

*Values are mean of three soil samples.*

*Different letters indicate significant differences (ANOVA, *P* < 0.05, Tukey’s HSD *post hoc* analysis) among tillage treatments.*

*Significant at 0.001 level.*

### Diversity of the Bacterial Community in the Sugarcane Rhizosphere

**FIGURE 1:**
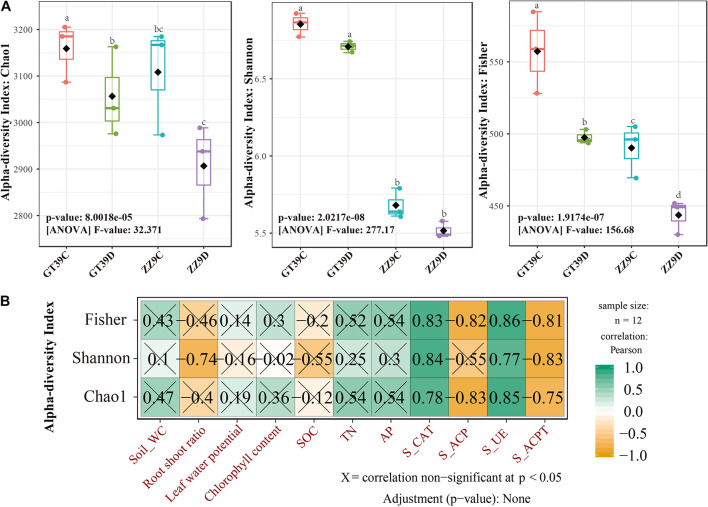
Analysis of differences in microbial diversity based on water treatment and variety differences. **(A)** Measured values of rhizobacteria alpha diversity in Chao 1 abundance, Shannon diversity, and Fisher among different water treatments and cultivars. **(B)** Analysis of the relationship between bacterial alpha diversity and environmental factors using Pearson Correlation.

Chao1, Shannon, and Fisher indices were used to analyze the α-diversity of sugarcane rhizosphere bacterial communities under the different treatments (Figure 1A). The results showed significant differences in rhizosphere bacterial diversity between ZZ9 and GT39 under control and drought treatments; the rhizosphere bacterial diversity was greater in the GT39 rhizosphere than in the ZZ9 rhizosphere. A significant difference in rhizosphere bacterial richness was observed between the control and drought treatments for GT39 but not for ZZ9. The Shannon index was significantly different between the two cultivars; however, there was no significant difference in the Shannon index for the different water treatments within the same cultivar. The Fisher index showed a significant difference among the four treatments, which indicated the presence of different species of rhizosphere bacteria in the four treatments. Pearson correlation analysis revealed a significant correlation between α-diversity and soil enzyme activities: there was a significant positive correlation with S-CAT and S-UE and a significant negative correlation with S-ACP and S-ACPT (Figure 1B).

**FIGURE 2:**
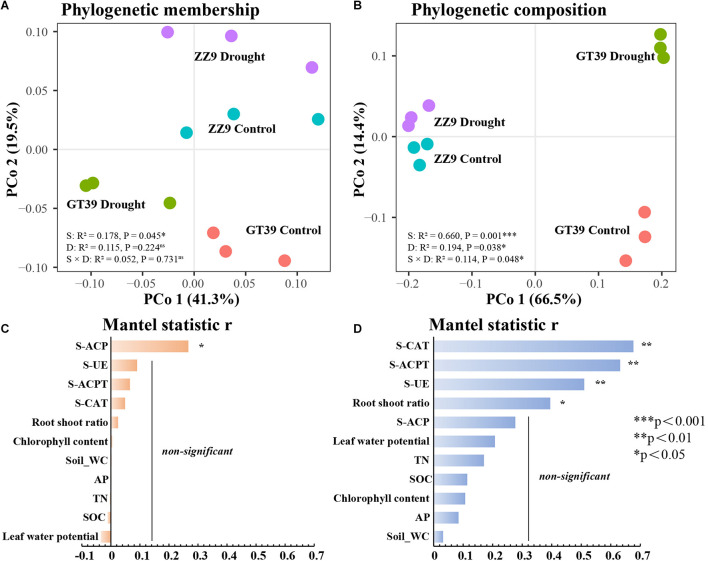
PCoA analysis of beta diversity based on Unifrac distance. **(A)** PCoA analysis of β diversity based on unweighted UinFrace distance, where PCo1 axis shows the effect of water treatment, and PCo2 represents the effect of variety differences on microbial community β diversity. **(B)** PCoA based on weighted UniFrac distance According to the analysis, the PCo1 axis shows the main influencing factors, and the PCo2 represents the effect of water treatment on the microbial community β diversity. **(C,D)** Correlation between environmental factors and correlation between bacterial beta-diversity and environmental factors in two sugarcane cultivars using Mantel test. **(C)** Mantel statistic analysis of the correlation between β diversity of ZZ9 varieties and environmental factors. **(D)** Mantel statistic analysis of the correlation between β diversity of GT39 varieties and environmental factors.

Principal coordinates analysis (PCoA) based on the weighted (composition) and unweighted UniFrac (membership) distance was conducted for the rhizosphere bacterial communities in the different treatments (Figures 2A,B). The phylogenetic membership distribution of the rhizosphere bacterial communities in the four treatments was dispersed. ZZ9C and ZZ9D were less affected by drought stress, whereas GT39C and GT39D were distributed on both sides of the first principal coordinate (PCo1) and were significantly affected by drought stress. Variety had a stronger effect on the phylogenetic composition of the GT39 rhizosphere bacteria and a weaker effect on that of the ZZ9 rhizosphere bacteria. We analyzed the relationships between environmental factors and the bacterial community using the Mantel test (Figures 2C,D). The results showed that the phylogenetic membership of the sugarcane rhizosphere bacterial community was correlated only with S-ACP (*P* < 0.05), whereas the phylogenetic composition was significantly correlated with S-ACPT, S-CAT, and S-UE (*P* < 0.01).

### Effect of Drought Stress on the Bacterial Community of the Sugarcane Rhizosphere

**FIGURE 3:**
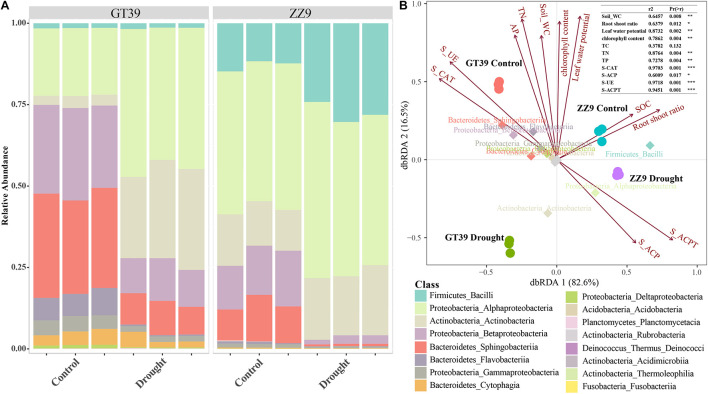
**(A)** A class-based population structure analysis (relative abundance) of the rhizosphere microbial bacterial communities of ZZ9 and GT39, and the main active populations are displayed. **(B)** The main active populations and environmental factors affecting the community are analyzed by dbRDA (environmental factors are represented by arrows).

A class-based population structure analysis of the rhizosphere bacteria under different treatments revealed the dominant microflora in the rhizosphere environment based on their relative abundance (Figure 3A). The main groups of sugarcane rhizosphere bacteria were Bacilli, Alphaproteobacteria, Actinobacteria, Betaproteobacteria, Sphingobacteriia, and Flavobacteria. In the control treatment, the species compositions of the rhizosphere bacterial communities of GT39 and ZZ9 were similar, but their abundances were quite different. Compared with that in GT39C, the relative abundance of Alphaproteobacteria and Actinobacteria in GT39D was significantly increased, whereas that of Betaproteobacteria, Sphingobacteriia, and Flavobacteria was significantly decreased. In ZZ9D, the relative abundances of Bacilli and Actinobacteria were increased, whereas the abundances of Betaproteobacteria and Sphingobacteriia were decreased. dbRDA analysis of the correlation between the major bacteria in the community and environmental factors (Figure 3B) revealed that drought stress accounted for 82.6% of the changes in bacterial flora, whereas the difference in drought tolerance of the two cultivars accounted for 16.5% of the changes. Bacteroidetes and Proteobacteria were closely related to S-UE and S-CAT, whereas Alphaproteobacteria was closely related to S-ACP and S-ACPT.

**FIGURE 4:**
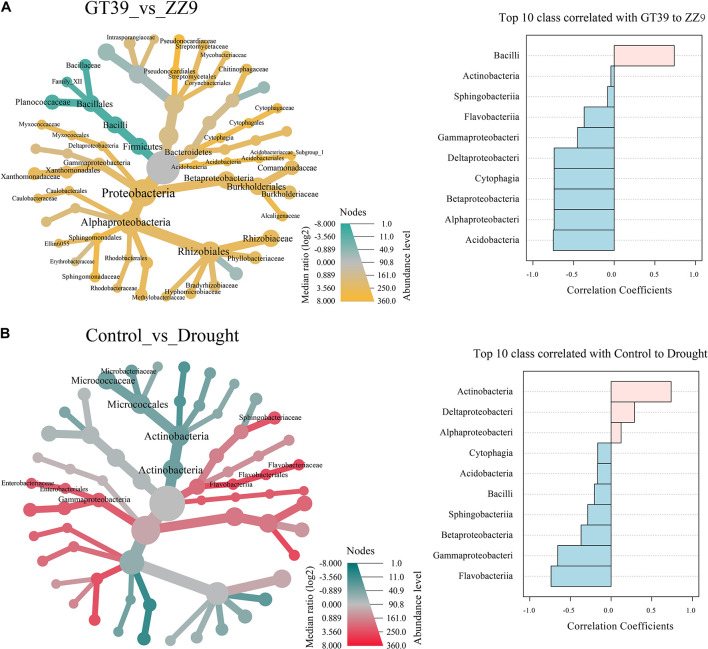
Visualization of classification differences in tree heat maps. **(A)** The influence of the differences between the GT39 and ZZ9 varieties on the rhizosphere flora. EdgeR represents the top ten major flora with a greater correlation with the drought tolerance of the two varieties, and pink and blue indicate the drought resistance of the variety. Relevance. **(B)** Control the differential effects of watering and drought stress on the rhizobacteria, edgeR represents the top ten major flora associated with water stress, pink and blue represent the correlation with the degree of water stress

The heat tree showed the effects of different variables (cultivar and drought stress) on the main bacterial flora, and edgeR revealed the bacterial groups that were most affected by the two variables (Figure 4). In the ZZ9 rhizosphere, Bacilli was the main group that responded to drought stress, whereas in the GT39 rhizosphere, Proteobacteria and Actinomycetes showed a marked response to drought stress. Comparison of the control and drought treatments revealed that Actinobacteria, Gammaproteobacteria, and Flavobacteria were mainly affected. Compared with the control, drought stress was positively correlated with the abundance of Actinobacteria, Deltaproteobacteria, and Alphaproteobacteria and negatively correlated with that of Gammaproteobacteria, Flavobacteriia, and other groups.

### Response of Drought-Tolerant Strains of Rhizosphere Bacteria to the Different Sugarcane Cultivars

The changes in the rhizosphere bacterial community related to drought resistance and environmental factors in the two sugarcane cultivars under drought conditions were assessed using MENA. To identify the factors that were strongly correlated with the rhizosphere bacterial community, we separately analyzed the modules with the most node connections. The modules that showed a high correlation of microbiota with environmental factors were Modules 15 and 18 (Supplementary Figures 3, 4). The summary points for GT39 (total nodes = 260, total links = 1750) were fewer than those for the drought-tolerant variety, ZZ9 (total nodes = 341, total links = 1908). However, the GT39 node centralization degree and density were higher than those for ZZ9 (Table 3). The GT39 (drought-sensitive) microbial community had a more complex degree of association (Table 3).

**TABLE 3:** Topological characteristics of two varieties Network analysis.

Network indexes	GT39-Network	ZZ9-Network
Total nodes	260	341
Total links	1750	1908
R square of power-law	0.645	0.706
Average degree (avgK)	13.462	11.191
Average clustering coefficient (avgCC)	0.419	0.406
Average path distance (GD)	4.145	6.003
Geodesic efficiency (E)	0.334	0.258
Harmonic geodesic distance (HD)	2.991	3.873
Maximal degree	55	59
Nodes with max degree	OTU_847;s-acp	s-acp
Centralization of degree (CD)	0.162	0.141
Maximal betweenness	2124.394	5634.936
Nodes with max betweenness	OTU_3435	OTU_751
Centralization of betweenness (CB)	0.055	0.088
Maximal stress centrality	139230	281349
Nodes with max stress centrality	OTU_433	OTU_177
Centralization of stress centrality (CS)	3.593	4.385
Maximal eigenvector centrality	0.179	0.183
Nodes with max eigenvector centrality	OTU_81	s-acp
Centralization of eigenvector centrality (CE)	0.146	0.159
Density (D)	0.052	0.033
Reciprocity	1	1
Transitivity (Trans)	0.596	0.587
Connectedness (Con)	0.685	0.667
Efficiency	0.929	0.955
Hierarchy	0	0
Lubness	1	1

**FIGURE 5:**
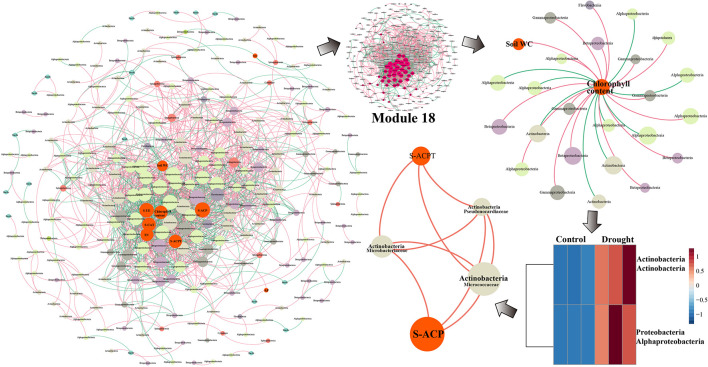
The network analysis of MENA shows that there is a significant interaction between the rhizobacteria community OUT under normal water and drought stress in the sugarcane variety GT39. The node indicates that the bacteria OUT in the microbial network with significant interactions are determined by the degree of connection. It is colored according to the system-level classification membership. The red line connecting the nodes indicates positive interaction, and the green line indicates negative interaction. Among them, GT39 (drought sensitive) mainly depends on Actinobacteria flora in response to drought stress Groups are closely related to S-ACP and S-ACPT.

The GT39 analysis further demonstrated that the environmental factors of soil water content, chlorophyll content, S-UE, S-ACP, S-CAT, S-ACPT, and TN were located in the center of the network and were closely connected to the bacterial flora (Figure 5). In Module 18, the main bacterial groups related to environmental factors were Alphaproteobacteria, Betaproteobacteria, Gammaproteobacteria, and Actinobacteria. Chlorophyll content showed a direct negative correlation with Alphaproteobacteria and Actinobacteria, whereas chlorophyll content and soil water content were positively correlated with Betaproteobacteria and Gammaproteobacteria (Figure 5). In the drought treatment, the abundance of Actinobacteria and Alphaproteobacteria changed significantly, and Actinobacteria were closely and positively correlated with S-ACPT and S-ACP.

**FIGURE 6:**
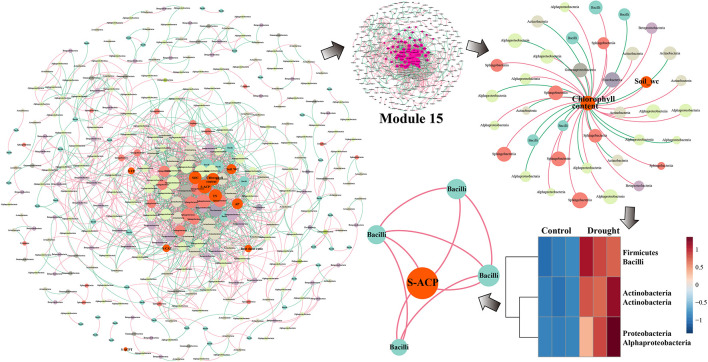
Network analysis of MENA, the analysis shows that there are different degrees of association between the bacterial communities of rhizosphere of sugarcane variety ZZ9. The nodes represent the bacteria OUT that have significant interaction in the microbial network, the size of which is determined by the degree of connection, and they are classified according to the system level. The relationship is colored. The red line connecting the nodes indicates positive interaction, and the green line indicates negative interaction. The rhizosphere bacterial community of the drought-tolerant variety ZZ9 mainly relies on the Bacilli flora (PGPR) to respond to drought stress. S-ACP is closely related.

In the network analysis of ZZ9, the environmental factors in the center were primarily soil water content, chlorophyll content, TN, S-ACP, AP, and SOC, whereas S-UE, S-CAT, and the root-shoot ratio were relatively close to the edge (Figure 6). The core key nodes in Module 15 were concentrated, and Bacilli, Actinobacteria, Alphaproteobacteria, Sphingobacteriia and others were highly connected to the environmental factors in the central region. The bacterial flora negatively correlated with chlorophyll content were Alphaproteobacteria, Actinobacteria, and Bacilli. The abundance of these three classes increased significantly in drought-treated ZZ9, and a close connecting line was observed between Bacilli and S-ACP, indicating a high degree of correlation.

## Discussion

### Drought Stress Reduced the Bacterial Diversity in the Sugarcane Rhizosphere

Differences in host plants and drought stress are two important factors affecting rhizosphere bacteria (Naylor et al., 2017). In the present study, we found that drought stress reduced the bacterial diversity in the rhizosphere of sugarcane, especially in the drought-sensitive cultivar GT39, and a significant correlation was detected between this change in bacterial diversity and soil enzyme activities (Figure 1). Drought stress stimulates the host plant response to a varying degree, depending on the drought tolerance of the host plant, which in turn affects the rhizosphere microorganisms (Larkunthod et al., 2018; de Vries et al., 2020). The phylogenetic membership and composition of the rhizosphere bacterial community of the drought-tolerant cultivar ZZ9 were more stable than those of the drought-sensitive cultivar GT39 (Figures 2A,B). The effect of cultivar type on rhizosphere microorganisms has been widely studied (Liljeroth and Bååth, 1988; Babu S. et al., 2015). Zhao et al., reported that sugarcane with strong stress resistance can resist drought and other stress conditions by utilizing rhizosphere microorganisms in an arid environment (Liu et al., 2020; Zhao et al., 2020b).

However, the rhizosphere is a complex environment occupied by microorganisms, which themselves are very sensitive to changes triggered by drought stress, including root exudates, soil conditions, and other factors (Wang et al., 2020). The rhizosphere soils of sugarcane cultivars with different drought tolerances differed significantly in nutrient composition and enzyme activities (Table 1). According to the Pearson’s correlation analysis, only soil enzyme activities were significantly correlated (*P* < 0.05) with the α-diversity of the rhizosphere bacterial community (Figure 1B). The deficiency in soil nutrients caused by drought stress is initiated by the decrease in the enzyme cycle rate, which is responsible for nutrient cycling and decomposition under drought stress (Hueso et al., 2012). He et al., showed that drought stress reduces TN in the soil, slows down the degradation of organophosphorus, and affects the absorption and release rates of C by plants (van der Molen et al., 2011; He and Dijkstra, 2014). Rhizosphere microorganisms maintain the soil nutrient supply by increasing the metabolic activity in the rhizosphere soil environment, promoting the effective mineralization of soil nutrients, and decomposing organic matter (Tarkka et al., 2008). S-ACP, S-UE, and S-ACPT are important enzymes involved in soil nutrient cycling (Shukla and Varma, 2010). Moreover, the changes in the bacterial communities in the sugarcane rhizosphere were significantly correlated with soil enzyme activities (Figures 2C,D). Therefore, we concluded that the bacterial community of drought-tolerant sugarcane cultivars is more stable under drought stress, whereas the rhizosphere bacterial community of drought-sensitive sugarcane cultivars is less stable and that this is significantly correlated with soil enzyme activities.

### Differences in the Drought Tolerance of Sugarcane Cultivars Led to Different Core Drought-Resistant Bacterial Strains

The response of the rhizosphere bacterial community to stress is closely related to the adaptability of plants to adversity (Gao et al., 2019). Analysis of the relative abundance of 16S rRNA in the rhizosphere revealed similar species compositions but significantly different relative abundances of different bacterial strains between the drought-tolerant ZZ9 cultivar and the drought-sensitive GT39 cultivar (Figure 3). A previous study by Kalinowski et al., showed that plant adaptation to stress can change the bacterial composition of the rhizosphere (Kalinowski and Halden, 2012), which was confirmed in our experiments. Under drought stress, the relative abundances of Alphaproteobacteria and Actinobacteria significantly increased in the water-sensitive cultivar, whereas it was already high in the drought-resistant cultivar under control conditions. Exposure to drought stress increased the abundance of Bacilli (Figure 3A). We conclude that the rhizosphere bacteria with increased relative abundance under drought stress are usually those with some level of drought tolerance and are able to maintain the rhizosphere environment under stress (Naylor and Coleman-Derr, 2018; Yasmin et al., 2021). The changes in Alphaproteobacteria and Actinobacteria abundance were positively correlated with drought stress, whereas the increase in the relative abundance of Bacilli had a positive correlation with the drought tolerance attributes of the drought-tolerant variety ZZ9 (Figure 4). A previous study found that Alphaproteobacteria can make full use of sugarcane rhizosphere secretions under environmental stress to improve the rhizosphere environment, and this class dominates the rhizosphere community of sugarcane (Da Costa et al., 2018). Actinomycetes and Bacilli are known plant growth-promoting bacteria that boost the adaptation of host plants under drought stress (Naylor et al., 2017). A rich community of drought-resistant bacteria exist in the rhizosphere of the drought-tolerant sugarcane cultivar under optimal soil conditions, and the abundance of these bacteria increased in the rhizosphere of drought-sensitive GT39 under drought stress.

Drought stress reduced the activities of various tested soil enzymes, such as S-ACP, S-UE, and enzymes involved in the N cycle, and it reduced the nutrient supply of plants (Sardans and Peñuelas, 2005). Rhizosphere bacteria can stimulate the decomposition of organic matter; enhance the solubility of C, N, and P; and promote the growth of host plant roots (Dijkstra et al., 2013; Li et al., 2013). We believe that there is a close correlation between rhizosphere bacteria and rhizosphere enzyme activities under drought stress owing to the interaction between the two, which promotes soil nutrient cycling (Figures 2D, 3B). For example, Da Costa et al., showed that members of Alphaproteobacteria could efficiently utilize the C in the rhizosphere exudates of sugarcane (Da Costa et al., 2018). Actinomycetes widely exist in soil ecosystems and can affect the decomposition and formation of soil humus to regulate the balance of soil nutrients (Yandigeri et al., 2012). *Bacillus*, a genus of plant growth-promoting bacteria, can induce nutrient absorption and the growth of host plants and stimulate the defense mechanisms of host plants under stress conditions (Sivasakthi et al., 2014). S-ACPT, S-ACP, S-UE, and other enzymes that are closely related to rhizosphere bacteria are key enzymes involved in the N and P cycles and soil protein decomposition (Cao et al., 2003). This explains the strong correlation between drought-responsive flora and soil physicochemical properties revealed by the dbRDA analysis (Figure 3B).

In conclusion, drought-resistant bacterial strains are dominant in the rhizosphere of drought-tolerant sugarcane cultivars, whereas the abundance of drought-tolerant bacteria in drought-sensitive sugarcane cultivars increases only when they are subjected to drought stress. Drought-tolerant bacteria, together with soil enzyme activities, maintain nutrient cycling in rhizosphere soil to ensure an adequate nutrient supply to sugarcane roots under drought stress.

### Response of Drought-Resistant Bacteria in the Sugarcane Rhizosphere to Drought Stress

Molecular ecological network analysis (MENA) was used to elucidate the ecological community structure and interactions among sugarcane rhizosphere bacteria under drought stress. Durán et al., found that the complexity of plant root microbial networks is related to plant survival, and the interaction with the rhizosphere microflora is key to the environmental adaptability of host plants (Durán et al., 2018). We found that the complexity of the sugarcane rhizosphere bacterial ecological network under drought stress was higher in the drought-tolerant sugarcane cultivar ZZ9 than in the drought-sensitive cultivar GT39 (Figures 5, 6 and Table 3). The high connectivity of the complex rhizosphere microflora network is the main factor that renders this network more resistant to environmental stress than a simple network environment (Santolini and Albert-László, 2018). In addition, the rhizosphere bacterial OTUs of the two cultivars showed different high density-associated regions under drought stress, and significantly more strains of rhizosphere bacteria were associated with drought-tolerant ZZ9 than with drought-sensitive GT39 (Supplementary Figures 3, 4). Therefore, we concluded that the difference in the rhizosphere bacterial community between the two cultivars is a factor that contributes to the difference in drought tolerance between the two sugarcane cultivars. However, the resistance of host plants to environmental stress is also an important factor affecting the structure of the rhizosphere bacterial community (Berendsen et al., 2012). It is because of this close correlation that abiotic environmental factors, such as drought stress, may affect both of them simultaneously (Wardle et al., 2004).

**FIGURE 7:**
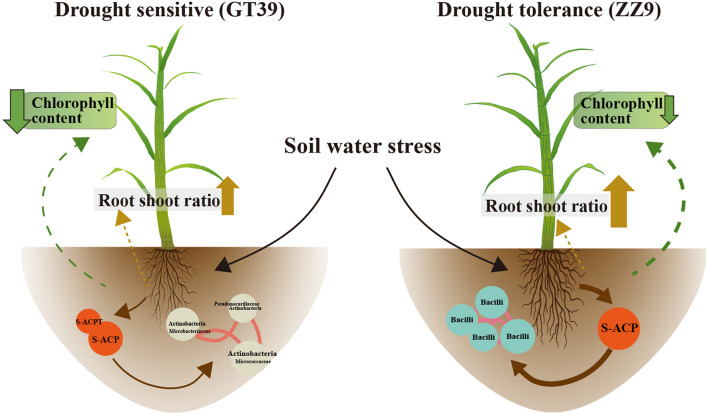
Changes in chlorophyll content and root exudates of the two sugarcane varieties GT39 and ZZ9 under water stress conditions and their interaction with the main rhizosphere bacterial communities.

The experimental results showed significant differences between GT39 and ZZ9 under drought stress, not only in the physiological characteristics of drought resistance (Table 2), but also in the response of the core drought-resistant bacteria (Figures 5, 6). The differences in the rhizosphere exudates of the host plants are the main driving force of the observed change in rhizosphere bacteria under drought stress (Li X. et al., 2019). The drought-responsive Actinobacteria of GT39 were positively correlated with S-ACP and S-ACPT (Figure 5), whereas Bacilli, which were the dominant rhizosphere bacteria of ZZ9 under drought stress, were positively correlated with S-ACP (Figure 6). As important elements of root exudates in the rhizosphere region, S-ACP and S-ACPT play important roles in the oxidative metabolism of soil nutrients, affecting the mineralization of phosphorus in rhizosphere soil and the transformation of soil protein components, respectively (Mijangos et al., 2006; Badri and Vivanco, 2009; Babu A. G. et al., 2015). Based on the close correlation between the components of the rhizosphere environment, we believe that the changes in rhizosphere exudates that occur in response to soil nutrient status under drought stress affect the response of drought-resistant rhizosphere bacteria (Canarini et al., 2016; Naylor and Coleman-Derr, 2018). In addition, many studies have shown that Actinobacteria can also effectively regulate the C and N contents in the soil under drought stress, maintain the health status of rhizosphere soil, and effectively alleviate abiotic stresses, such as salt, alkali, and drought (Badri and Vivanco, 2009). Bacilli are also believed to promote phosphate solubilization and are an important regulator of AP content in rhizosphere soil (Dias et al., 2009; Mujahid et al., 2015). Therefore, the drought resistance of sugarcane is achieved by both self-regulation and the response of drought-resistant bacteria in the rhizosphere. Drought-resistant bacteria affect soil nutrient cycling under the influence of root exudates, and both jointly regulate the nutrient supply of sugarcane root in water-deficient environments (Figure 7).

## Conclusion

We investigated the changes in the rhizosphere bacterial communities of two sugarcane cultivars with different drought resistance responses. This difference in drought resistance did not lead to significant differences in the rhizosphere bacterial community structure between the two sugarcane cultivars under drought stress. However, the diversity of the rhizosphere bacteria was decreased in both sugarcane cultivars under drought stress, and the change was greater in the rhizosphere of the drought-sensitive sugarcane cultivar than in the rhizosphere of the drought-resistant sugarcane cultivar. In addition, we found that the drought-tolerant cultivar was better adapted to drought owing to the high abundance of drought-resistant bacteria in its rhizosphere under normal water conditions, whereas the abundance of drought-resistant bacteria in the rhizosphere of the drought-sensitive cultivar began to increase only after exposure to drought stress. Thus, drought-sensitive cultivars require more time to respond to drought stress and are therefore more vulnerable to damage. The resistance of sugarcane to drought stress is related to both physiological adjustments and the regulation of rhizosphere bacteria. Under the influence of rhizosphere exudates, the core drought-resistant bacteria in the sugarcane rhizosphere regulate the nutrient status of rhizosphere soil and improve the drought resistance of sugarcane.

## Data Availability Statement

The datasets presented in this study can be found in online repositories. The names of the repository/repositories and accession number(s) can be found below: https://www.ncbi.nlm.nih.gov/, PRJNA655948.

## Author Contributions

QL, ZW, XZ, and YL contributed to the design of the experiments, data analysis, and manuscript writing. QL, SX, and YL contributed to the experimentation. QL and ZW contributed to the data interpretation. YP and WD participated in the revision of the article. YX, JD, and BW cultivated sugarcane growth during the experiment and participated in the determination of experimental indicators. All authors contributed to the article and approved the submitted version.

## Conflict of Interest

The authors declare that the research was conducted in the absence of any commercial or financial relationships that could be construed as a potential conflict of interest.

## Publisher’s Note

All claims expressed in this article are solely those of the authors and do not necessarily represent those of their affiliated organizations, or those of the publisher, the editors and the reviewers. Any product that may be evaluated in this article, or claim that may be made by its manufacturer, is not guaranteed or endorsed by the publisher.

## Footnotes

1
http://www.usadellab.org/cms/?page=trimmomatic

2
https://ccb.jhu.edu/software/FLASH/

3
http://www.mothur.org

4
https://www.drive5.com/usearch/

5
https://www.ncbi.nlm.nih.gov/sra

6
http://ieg4.rccc.ou.edu/mena/

## References

[B1] Aimin Z. Liu E. Liu J. Feng S. Gong S. Wang J. (2018). Characterization of increased cuticular wax mutant and analysis of genes involved in wax biosynthesis in Dianthus spiculifolius. *Hortic. Res.* 5:40. 10.1038/s41438-018-0044-z PMC6068182 30083355

[B2] Andreo-Jimenez B. Vandenkoornhuyse P. Van A. L. Heutinck A. Bouwmeester H. (2019). Plant host and drought shape the root associated fungal microbiota in rice. *PeerJ* 7:e7463. 10.7717/peerj.7463 31565550 PMC6744933

[B3] Babu A. G. Kim S. W. Yadav D. R. Hyum U. Adhikari M. Lee Y. S. (2015). *Penicillium menonorum*: a novel fungus to promote growth and nutrient management in cucumber plants. *Mycobiology* 43 49–56. 10.5941/myco.2015.43.1.49 25892915 PMC4397380

[B4] Babu S. Bidyarani N. Chopra P. Monga D. Kumar R. Prasanna R. (2015). Evaluating microbe-plant interactions and varietal differences for enhancing biocontrol efficacy in root rot disease challenged cotton crop. *Eur. J. Plant Pathol.* 142 345–362. 10.1007/s10658-015-0619-6

[B5] Badri D. V. Vivanco J. M. (2009). Regulation and function of root exudates. *Plant Cell Environ.* 32 666–681. 10.1111/j.1365-3040.2009.01926.x 19143988

[B6] Bao S. (2000). *Soil and agricultural chemistry analysis.* Beijing: China agriculture press.

[B7] Benitez M.-S. Osborne S. L. Lehman R. M. (2017). Previous crop and rotation history effects on maize seedling health and associated rhizosphere microbiome. *Sci. Rep.* 7 1–13. 29146930 10.1038/s41598-017-15955-9 PMC5691165

[B8] Berendsen R. L. Pieterse C. M. J. Bakker P. (2012). The rhizosphere microbiome and plant health. *Trends Plant Sci.* 17 478–486. 10.1016/j.tplants.2012.04.001 22564542

[B9] Buchanan B. B. Gruissem W. Jones R. L. (2015). *Biochemistry and Molecular Biology of Plants.* Hoboken, NJ: John Wiley & Sons.

[B10] Butt H. Piatek A. Li L. Reddy A. S. N. Mahfouz M. M. (2019). Multiplex CRISPR mutagenesis of the serine/arginine-rich (SR) gene family in rice. *Genes* 10:596. 10.3390/genes10080596 31394891 PMC6723545

[B11] Canarini A. Andrew M. Dijkstra F. A. (2016). Drought effects on *Helianthus annuus* and Glycine max metabolites: from phloem to root exudates. *Rhizosphere* 2 85–97. 10.1016/j.rhisph.2016.06.003

[B12] Cao H. Sun H. Yang H. Sun B. Zhao Q. (2003). A review: soil enzyme activity and its indication for soil quality. *Chinese J. Appl. Environ. Biol.* 9 105–109.

[B13] Cao M.-J. Zhang Y.-L. Liu X. Huang H. Zhou X. E. Wang W.-L. (2017). Combining chemical and genetic approaches to increase drought resistance in plants. *Nat. Commun.* 8 1–12. 29084945 10.1038/s41467-017-01239-3 PMC5662759

[B14] Caporaso J. G. Kuczynski J. Stombaugh J. Bittinger K. Bushman F. D. Costello E. K. (2010). QIIME allows analysis of high-throughput community sequencing data. *Nat. Methods* 7 335–336. 20383131 10.1038/nmeth.f.303 PMC3156573

[B15] Chaves M. M. Pereira J. S. Maroco J. Rodrigues M. L. Ricardo C. P. P. Osório M. L. (2002). How plants cope with water stress in the field? Photosynthesis and growth. *Ann. Bot.* 89 907–916. 10.1093/aob/mcf105 12102516 PMC4233809

[B16] Da Costa D. P. Dias A. C. Cotta S. R. Vilela D. De Andrade P. A. Pellizari V. H. (2018). Changes of bacterial communities in the rhizosphere of sugarcane under elevated concentration of atmospheric CO 2. *GCB Bioenergy* 10 137–145. 10.1111/gcbb.12476

[B17] de Vries F. T. Griffiths R. I. Knight C. G. Nicolitch O. Williams A. (2020). Harnessing rhizosphere microbiomes for drought-resilient crop production. *Science* 368 270–274. 10.1126/science.aaz5192 32299947

[B18] Demers J. E. Gugino B. K. Jimenez-Gasco M. D. M. (2015). Highly diverse endophytic and soil fusarium oxysporum populations associated with field-grown tomato plants. *Appl. Environ. Microbiol.* 81 81–90. 10.1128/aem.02590-14 25304514 PMC4272710

[B19] Dias A. C. Costa F. E. Andreote F. D. Lacava P. T. Teixeira M. A. Assumpçao L. C. (2009). Isolation of micropropagated strawberry endophytic bacteria and assessment of their potential for plant growth promotion. *World J. Microbiol. Biotechnol.* 25 189–195. 10.1007/s11274-008-9878-0

[B20] Dijkstra F. A. Carrillo Y. Pendall E. Morgan J. A. (2013). Rhizosphere priming: a nutrient perspective. *Front. Microbiol.* 4:216. 10.3389/fmicb.2013.00216 23908649 PMC3725428

[B21] Durán P. Thiergart T. Garrido-Oter R. Agler M. Kemen E. Schulze-Lefert P. (2018). Microbial interkingdom interactions in roots promote *Arabidopsis* survival. *Cell* 175 973.e–983.e. 30388454 10.1016/j.cell.2018.10.020 PMC6218654

[B22] Edgar R. C. (2010). Search and clustering orders of magnitude faster than BLAST. *Bioinformatics* 26 2460–2461. 10.1093/bioinformatics/btq461 20709691

[B23] Gao J. Luo Y. Wei Y. Huang Y. Zhang H. He W. (2019). Effect of aridity and dune type on rhizosphere soil bacterial communities of *Caragana microphylla* in desert regions of northern China. *PLoS one* 14:e0224195. 10.1371/journal.pone.0224195 31626675 PMC6799922

[B24] Garsmeur O. Droc G. Antonise R. Grimwood J. Potier B. Aitken K. (2018). A mosaic monoploid reference sequence for the highly complex genome of sugarcane. *Nat. Commun.* 9 1–10. 29980662 10.1038/s41467-018-05051-5 PMC6035169

[B25] Gentile A. Dias L. I. Mattos R. S. Ferreira T. S. H. Menossi M. (2015). MicroRNAs and drought responses in sugarcane. *Front. Plant Ence* 6:1–13. 10.3389/fpls.2015.00058 25755657 PMC4337329

[B26] Ghannoum O. (2016). How can we breed for more water use-efficient sugarcane? *J. Exp. Bot.* 67 557–559. 10.1093/jxb/erw009 26839219 PMC5873515

[B27] Giehl R. F. von Wirén N. (2014). Root nutrient foraging. *Plant Physiol.* 166 509–517. 10.1104/pp.114.245225 25082891 PMC4213083

[B28] Griffiths C. A. Paul M. J. (2017). Targeting carbon for crop yield and drought resilience. *J. Sci. Food Agric.* 97 4663–4671. 10.1002/jsfa.8501 28653336 PMC5655914

[B29] Gupta B. Huang B. (2014). Mechanism of salinity tolerance in plants: physiological, biochemical, and molecular characterization. *Int. J. Genom.* 2014:701596. 10.1155/2014/701596 PMC3996477 24804192

[B30] Hajar-Azhari S. Wan-Mohtar W. A. A. Q. I Ab Kadir S. Abd Rahim M. H. Saari N. (2018). Evaluation of a Malaysian soy sauce koji strain Aspergillus oryzae NSK for γ-aminobutyric acid (GABA) production using different native sugars. *Food Sci. Biotechnol.* 27 479–488. 30263772 10.1007/s10068-017-0289-6 PMC6049641

[B31] He M. Dijkstra F. A. (2014). Drought effect on plant nitrogen and phosphorus: a meta-analysis. *New Phytol.* 204 924–931. 10.1111/nph.12952 25130263

[B32] Hilker M. Schwachtje J. Baier M. Balazadeh S. Bäurle I. Geiselhardt S. (2016). Priming and memory of stress responses in organisms lacking a nervous system. *Biol. Rev.* 91 1118–1133. 10.1111/brv.12215 26289992

[B33] Howard A. Comber S. Kifle D. Antai E. Purdie D. (1995). Arsenic speciation and seasonal changes in nutrient availability and micro-plankton abundance in Southampton water, UK. *Estuar. Coast. Shelf Sci.* 40 435–450. 10.1006/ecss.1995.0030

[B34] Hueso S. García C. Hernández T. (2012). Severe drought conditions modify the microbial community structure, size and activity in amended and unamended soils. *Soil Biol. Biochem.* 50 167–173. 10.1016/j.soilbio.2012.03.026

[B35] Iquebal M. A. Sharma P. Jasrotia R. S. Jaiswal S. Kaur A. Saroha M. (2019). RNAseq analysis reveals drought-responsive molecular pathways with candidate genes and putative molecular markers in root tissue of wheat. *Sci. Rep.* 9 1–18. 31558740 10.1038/s41598-019-49915-2 PMC6763491

[B36] Jin X. Yunzeng Z. Pengfan Z. Riera N. (2018). The structure and function of the global citrus rhizosphere microbiome. *Nat. Commun.* 9:4894. 10.1038/s41467-018-07343-2 PMC6244077 30459421

[B37] Jordan V. Yvan M. N. L. Audrey D. Maximilien G. A. M. Daniel M. Claire P. C. (2016). Fluorescent *Pseudomonas* strains with only few plant-beneficial properties are favored in the maize rhizosphere. *Front. Plant Sci.* 7:1212. 10.3389/fpls.2016.01212 27610110 PMC4996994

[B38] Jurburg S. D. Nunes I. Stegen J. C. Roux X. L. Priemé A. Sørensen S. J. (2017). Autogenic succession and deterministic recovery following disturbance in soil bacterial communities. *Sci. Rep.* 7:45691. 10.1038/srep45691 PMC5382530 28383027

[B39] Kalinowski T. Halden R. U. (2012). Can stress enhance phytoremediation of polychlorinated biphenyls? *Environ. Eng. Sci.* 29 1047–1052. 10.1089/ees.2012.0089 23236249 PMC3516413

[B40] Kavamura V. N. Taketani R. G. Lançoni M. D. Andreote F. D. Mendes R. de Melo I. S. (2013). Water regime influences bulk soil and rhizosphere of *Cereus jamacaru* bacterial communities in the Brazilian caatinga biome. *PLoS One* 8:e73606. 10.1371/journal.pone.0073606 24069212 PMC3775785

[B41] Khan N. Bano A. M. Babar A. (2020). Impacts of plant growth promoters and plant growth regulators on rainfed agriculture. *PLoS One* 15:e0231426. 10.1371/journal.pone.0231426 32271848 PMC7145150

[B42] Koech R. K. Malebe P. M. Nyarukowa C. Mose R. Kamunya S. M. Joubert F. (2019). Functional annotation of putative QTL associated with black tea quality and drought tolerance traits. *Sci. Rep.* 9 1–11. 30728388 10.1038/s41598-018-37688-z PMC6365519

[B43] Krause S. M. Meima-Franke M. Veraart A. J. Ren G. Ho A. Bodelier P. L. (2018). Environmental legacy contributes to the resilience of methane consumption in a laboratory microcosm system. *Sci. Rep.* 8 1–9. 29892072 10.1038/s41598-018-27168-9 PMC5995846

[B44] Kundariya H. Yang X. Morton K. Sanchez R. Axtell M. J. Hutton S. F. (2020). MSH1-induced heritable enhanced growth vigor through grafting is associated with the RdDM pathway in plants. *Nat. Commun.* 11 1–14. 33093443 10.1038/s41467-020-19140-x PMC7582163

[B45] Larkunthod P. Nounjan N. Siangliw J. L. Toojinda T. Sanitchon J. Jongdee B. (2018). Physiological responses under drought stress of improved drought-tolerant rice lines and their parents. *Notulae Bot. Horti Agrobotanici Cluj Napoca* 46 679–687. 10.15835/nbha46211188

[B46] Lee S. A. Kim Y. Kim J. M. Chu B. Joa J.-H. Sang M. K. (2019). A preliminary examination of bacterial, archaeal, and fungal communities inhabiting different rhizocompartments of tomato plants under real-world environments. *Sci. Rep.* 9 1–15. 31243310 10.1038/s41598-019-45660-8 PMC6594962

[B47] Li P. Wu H. Jin Y. Huang X. Chen Y. Yang X. (2019). Exploring the diversity and dynamic of bacterial community vertically distributed in tongguling national nature reserve in Hainan Island, China. *Braz. J. Microbiol.* 50 729–737. 10.1007/s42770-019-00078-2 31104215 PMC6863314

[B48] Li X. Jousset A. de Boer W. Carrión V. J. Zhang T. Wang X. (2019). Legacy of land use history determines reprogramming of plant physiology by soil microbiome. *ISME J.* 13 738–751. 10.1038/s41396-018-0300-0 30368524 PMC6461838

[B49] Li X. Mu Y. Cheng Y. Liu X. Nian H. (2013). Effects of intercropping sugarcane and soybean on growth, rhizosphere soil microbes, nitrogen and phosphorus availability. *Acta Physiol. Plant.* 35 1113–1119. 10.1007/s11738-012-1148-y

[B50] Li Y. Hu Y. Song D. Liang S. Qin X. Siddique K. H. (2019). The effects of straw incorporation with plastic film mulch on soil properties and bacterial community structure on the loess plateau. *Eur. J. Soil Sci.* 72 979–994. 10.1111/ejss.12912

[B51] Liljeroth E. Bååth E. (1988). Bacteria and fungi on roots of different barley varieties (*Hordeum vulgare* L.). *Biol. Fertil. Soils* 7 53–57.

[B52] Liu Y. Yang H. Liu Q. Zhao X. Xie S. Wang Z. (2020). Effect of two different sugarcane cultivars on rhizosphere bacterial communities of sugarcane and soybean upon intercropping. *Front. Microbiol.* 11:596472. 10.3389/fmicb.2020.596472 33519733 PMC7841398

[B53] Luo M. Liang J. (2005). Response of sugarcane to drought stress at seedling stage. *J. Trop. Crops* 026 38–41.

[B54] Meijuan Z. Yongjia Z. Shijie C. Yong D. (2018). Deciphering the bacterial composition in the rhizosphere of *Baphicacanthus cusia* (NeeS) Bremek. *Sci. Rep.* 8:15831. 10.1038/s41598-018-34177-1 PMC6202335 30361644

[B55] Mijangos I. Pérez R. Albizu I. Garbisu C. (2006). Effects of fertilization and tillage on soil biological parameters. *Enzyme Microb. Technol.* 40 100–106. 10.1016/j.enzmictec.2005.10.043

[B56] Mujahid T. Y. Subhan S. A. Wahab A. Masnoon J. Ahmed N. Abbas T. (2015). Effects of different physical and chemical parameters on phosphate solubilization activity of plant growth promoting bacteria isolated from indigenous soil. *J. Pharm. Nutr. Sci.* 5 64–70. 10.6000/1927-5951.2015.05.01.10

[B57] Navid B. Chantal H. Yantai G. Bunyamin T. A. Joan D. K. (2015). Genotype-specific variation in the structure of root fungal communities is related to chickpea plant productivity. *Appl. Environ. Microbiol.* 81 2368–2377. 10.1128/aem.03692-14 25616789 PMC4357931

[B58] Naylor D. Coleman-Derr D. (2018). Drought stress and root-associated bacterial communities. *Front. Plant Sci.* 8:2223. 10.3389/fpls.2017.02223 29375600 PMC5767233

[B59] Naylor D. DeGraaf S. Purdom E. Coleman-Derr D. (2017). Drought and host selection influence bacterial community dynamics in the grass root microbiome. *ISME J.* 11 2691–2704. 10.1038/ismej.2017.118 28753209 PMC5702725

[B60] Newman M. E. (2006). Modularity and community structure in networks. *Proc. Natl. Acad. Sci. U.S.A.* 103 8577–8582. 16723398 10.1073/pnas.0601602103 PMC1482622

[B61] Oksanen J. Blanchet F. G. Kindt R. Legendre P. Minchin P. O’hara R. (2013). *Community Ecology Package. R package version*, *2.0-2.*

[B62] Pascale A. Proietti S. I Pantelides S. Stringlis I. A. (2020). Modulation of the root microbiome by plant molecules: the basis for targeted disease suppression and plant growth promotion. *Front. Plant Sci.* 10:1741. 10.3389/fpls.2019.01741 32038698 PMC6992662

[B63] Peiffer J. A. Spor A. Koren O. Jin Z. Tringe S. G. Dangl J. L. (2013). Diversity and heritability of the maize rhizosphere m rhizosphere microbiome under field conditions. *Proc. Natl. Acad. Sci. U.S.A.* 110 6548–6553. 10.1073/pnas.1302837110 23576752 PMC3631645

[B64] Qin Q. Zhu J. Shi Z. Yu T. Cao K. (2017). Association of leaf anatomical structure characteristics with photosynthetic capacity and drought tolerance in seven sugarcane varieties. *Acta Plant Physiol.* 053 705–712.

[B65] Robinson M. D. McCarthy D. J. Smyth G. K. (2010). edgeR: a bioconductor package for differential expression analysis of digital gene expression data. *Bioinformatics* 26 139–140. 10.1093/bioinformatics/btp616 19910308 PMC2796818

[B66] Sade N. Gebretsadik M. Seligmann R. Schwartz A. Wallach R. Moshelion M. (2010). The role of tobacco Aquaporin1 in improving water use efficiency, hydraulic conductivity, and yield production under salt stress. *Plant Physiol.* 152 245–254. 10.1104/pp.109.145854 19939947 PMC2799360

[B67] Santolini M. Albert-László B. (2018). Predicting perturbation patterns from the topology of biological networks. *Proc. Natl. Acad. Sci. U.S.A.* 115 E6375–E6383. 29925605 10.1073/pnas.1720589115 PMC6142275

[B68] Sardans J. Peñuelas J. (2005). Drought decreases soil enzyme activity in a Mediterranean *Quercus ilex* L. forest. *Soil Biol. Biochem.* 37 455–461. 10.1016/j.soilbio.2004.08.004

[B69] Schimel J. Balser T. C. Wallenstein M. (2007). Microbial stress-response physiology and its implications for ecosystem function. *Eology* 88 1386–1394. 10.1890/06-0219 17601131

[B70] Shannon P. Markiel A. Ozier O. Baliga N. S. Wang J. T. Ramage D. (2003). Cytoscape: a software environment for integrated models of biomolecular interaction networks. *Genome Res.* 13 2498–2504. 10.1101/gr.1239303 14597658 PMC403769

[B71] Shao W. Li G. (2016). Research progress of soil enzymes function and its determination method. *Northern Hortic.* 000 188–193.

[B72] Shukla G. Varma A. (2010). *Soil Enzymology.* Berlin: Springer Science & Business Media.

[B73] Siraree A. Banerjee N. Kumar S. Khan M. Singh P. Sharma S. (2017). Identification of marker-trait associations for morphological descriptors and yield component traits in sugarcane. *Physiol. Mol. Biol. Plants* 23 185–196. 10.1007/s12298-016-0403-x 28250594 PMC5313407

[B74] Sivasakthi S. Usharani G. Saranraj P. (2014). Biocontrol potentiality of plant growth promoting bacteria (PGPR)-*Pseudomonas* fluorescens and *Bacillus subtilis*: a review. *Afr. J. Agric. Res.* 9 1265–1277.

[B75] Tarkka M. Schrey S. Hampp R. (2008). “Plant associated soil micro-organisms,” in *Molecular Mechanisms of Plant and Microbe Coexistence*, eds Nautiyal C. S. Dion P. (Berlin: Springer), 3–51. 10.1007/978-3-540-75575-3_1

[B76] Teramoto S. Takayasu S. Kitomi Y. Arai-Sanoh Y. Tanabata T. Uga Y. (2020). High-throughput three-dimensional visualization of root system architecture of rice using X-ray computed tomography. *Plant Methods* 16 1–14. 32426023 10.1186/s13007-020-00612-6 PMC7216661

[B77] Tisdall J. (2001). *Beginning Perl for Bioinformatics: An Introduction to Perl for Biologists.* Sebastopol, CA: O’Reilly Media, Inc.

[B78] Treseder K. K. (2008). Nitrogen additions and microbial biomass: a meta-analysis of ecosystem studies. *Ecol. Lett.* 11 1111–1120. 10.1111/j.1461-0248.2008.01230.x 18673384

[B79] van der Molen M. K. Dolman A. J. Ciais P. Eglin T. Gobron N. Law B. E. (2011). Drought and ecosystem carbon cycling. *Agric. For. Meteorol.* 151 765–773.

[B80] Verma A. Niranjana M. Jha S. Mallick N. Agarwal P. (2020). QTL detection and putative candidate gene prediction for leaf rolling under moisture stress condition in wheat. *Sci. Rep.* 10 1–13. 33122772 10.1038/s41598-020-75703-4 PMC7596552

[B81] Waldrop M. Firestone M. (2006). Response of microbial community composition and function to soil climate change. *Microb. Ecol.* 52 716–724. 10.1007/s00248-006-9103-3 17061172

[B82] Wang L. Xiang L. I. Tang S. Huang H. Jing Y. Tan F. (2013). Characteristic study of a new sugarcane variety GT39. *Agric. Sci. Technol.* 14 1550–1553.

[B83] Wang S. Cheng J. Liao Y. (2020). Response of Rhizosphere microecology to plants, soil and microbes. *Acta Microsc.* 29:43150.

[B84] Wardle D. A. Bardgett R. D. Klironomos J. N. Setälä H. Van Der Putten W. H. Wall D. H. (2004). Ecological linkages between aboveground and belowground biota. *Science* 304 1629–1633. 10.1126/science.1094875 15192218

[B85] Wei T. Simko V. Levy M. Xie Y. Jin Y. Zemla J. (2017). Package ‘corrplot’. *Statistician* 56:e24.

[B86] Welsh D. (2000). Ecological significance of compatible solute accumulation by micro-organisms: from single cells to global climate. *FEMS Microbiol. Rev.* 24 263–290. 10.1111/j.1574-6976.2000.tb00542.x 10841973

[B87] Wen T. Zhao M. Liu T. Huang Q. Yuan J. Shen Q. (2020). High abundance of Ralstonia solanacearum changed tomato rhizosphere microbiome and metabolome. *BMC Plant Biol.* 20:166. 10.1186/s12870-020-02365-9 32293273 PMC7160980

[B88] Yandigeri M. S. Meena K. K. Singh D. Malviya N. Singh D. P. Solanki M. K. (2012). Drought-tolerant endophytic actinobacteria promote growth of wheat (*Triticum aestivum*) under water stress conditions. *Plant Growth Regul.* 68 411–420. 10.1007/s10725-012-9730-2

[B89] Yao J. Sun D. Cen H. Xu H. Weng H. Yuan F. (2018). Phenotyping of *Arabidopsis* drought stress response using kinetic chlorophyll fluorescence and multicolor fluorescence imaging. *Front. Plant Sci.* 9:603. 10.3389/fpls.2018.00603 29868063 PMC5958224

[B90] Yasmin H. Bano A. Wilson N. L. Nosheen A. Naz R. Hassan M. N. (2021). Drought tolerant *Pseudomonas* sp. showed differential expression of stress-responsive genes and induced drought tolerance in *Arabidopsis thaliana*. *Physiol. Plant.* [Epub ahead of print]. 10.1111/ppl.13497 34245030

[B91] Yasmin H. Rashid U. Hassan M. N. Nosheen A. Naz R. Ilyas N. (2020). Volatile organic compounds produced by *Pseudomonas* pseudoalcaligenes alleviated drought stress by modulating defence system in Maize (*Zea mays* L.). *Physiol. Plant.* 172 896–911. 10.1111/ppl.13304 33314151

[B92] Yin S. Contest W. Xiaoyu H. Wenyan L. Wenlan L. Baoshan C. (2020). Response of new series of mid-cane varieties to drought stress and evaluation of drought resistance. *S. Agric. J.* 51:200.

[B93] Yu G. Smith D. K. Zhu H. Guan Y. Lam T. T. Y. (2017). ggtree: an R package for visualization and annotation of phylogenetic trees with their covariates and other associated data. *Methods Ecol. Evol.* 8 28–36. 10.1111/2041-210x.12628

[B94] Zhao P. Liu P. Yuan G. Jia J. Li X. Qi D. (2016). New insights on drought stress response by global investigation of gene expression changes in Sheepgrass (*Leymus chinensis*). *Front. Plant Sci.* 7:954. 10.3389/fpls.2016.00954 27446180 PMC4928129

[B95] Zhao X. Liu Q. Xie S. Jiang Y. Yang H. Wang Z. (2020b). Response of soil fungal community to drought-resistant Ea-DREB2B transgenic sugarcane. *Front. Microbiol.* 11:2329. 10.3389/fmicb.2020.562775 PMC7530946 33072024

[B96] Zhao X. Jiang Y. Liu Q. Yang H. Wang Z. Zhang M. (2020a). Effects of drought-tolerant Ea-DREB2B transgenic sugarcane on bacterial communities in soil. *Front. Microbiol.* 11:704. 10.3389/fmicb.2020.00704 PMC7214759 32431674

