# Supplementary material for: Drought Sensitivity of Sugarcane Cultivars Shapes Rhizosphere Bacterial Community Patterns in Response to Water Stress
Source: Front Microbiol. 2021 Oct 21;12:732989. doi: 10.3389/fmicb.2021.732989 (PMC8568056; doi:10.3389/fmicb.2021.732989)

Supplementary Materials

Supplementary Fig.1 Statistics of soil water content in different treatments

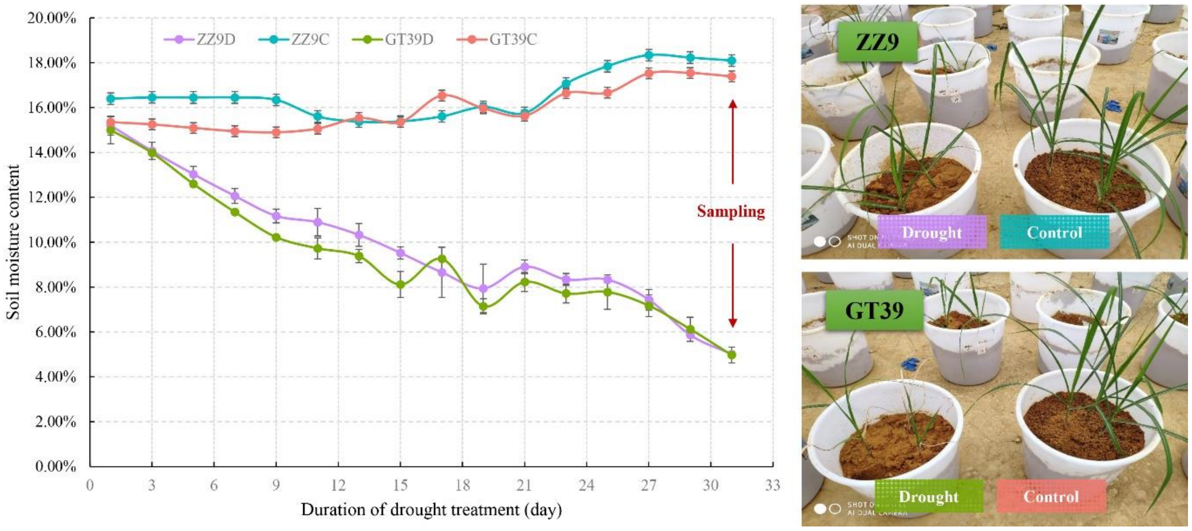

Supplementary Fig.2 Schematic diagram of Sample collection

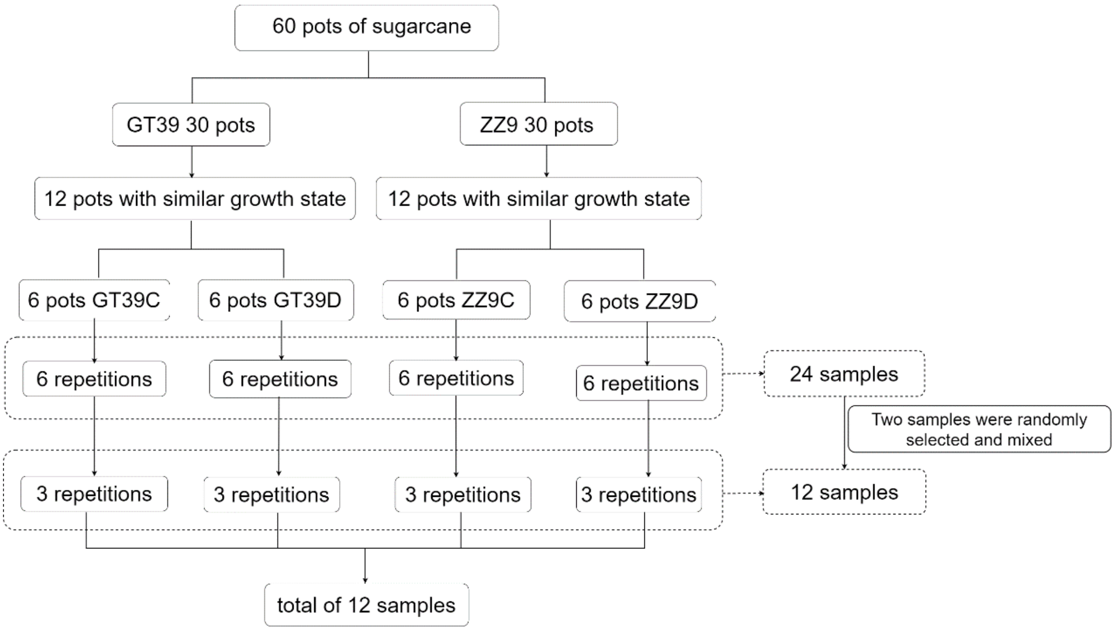

Supplementary Fig.3 ZZ9 rhizosphere bacteria OTUs module aggregation number statistics

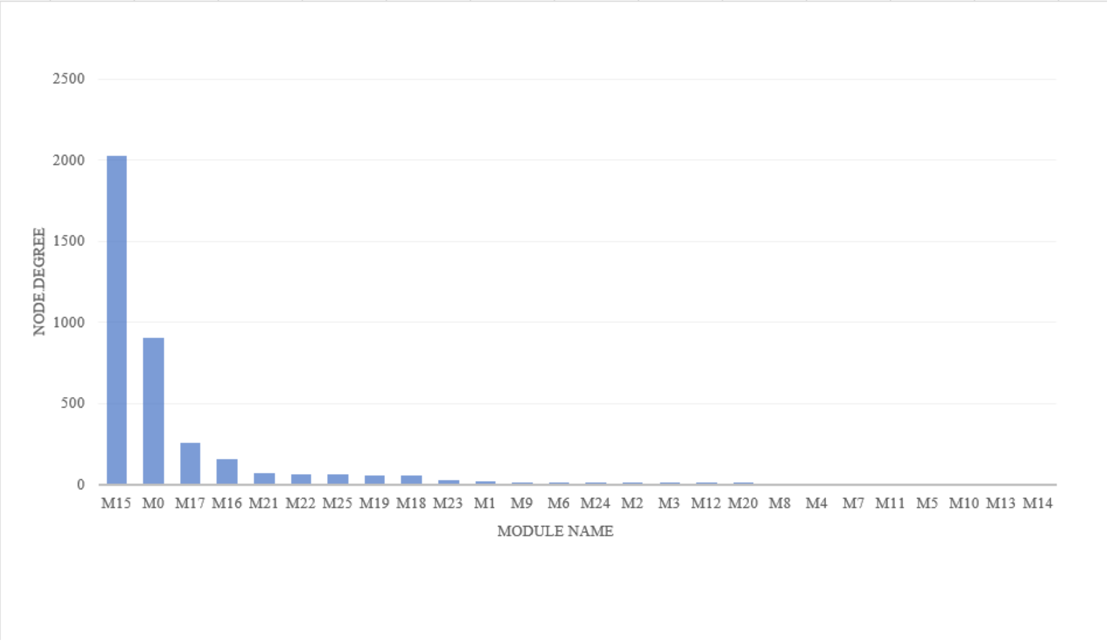

Supplementary Fig.4 GT39 rhizosphere bacteria OTUs module aggregation number statistics

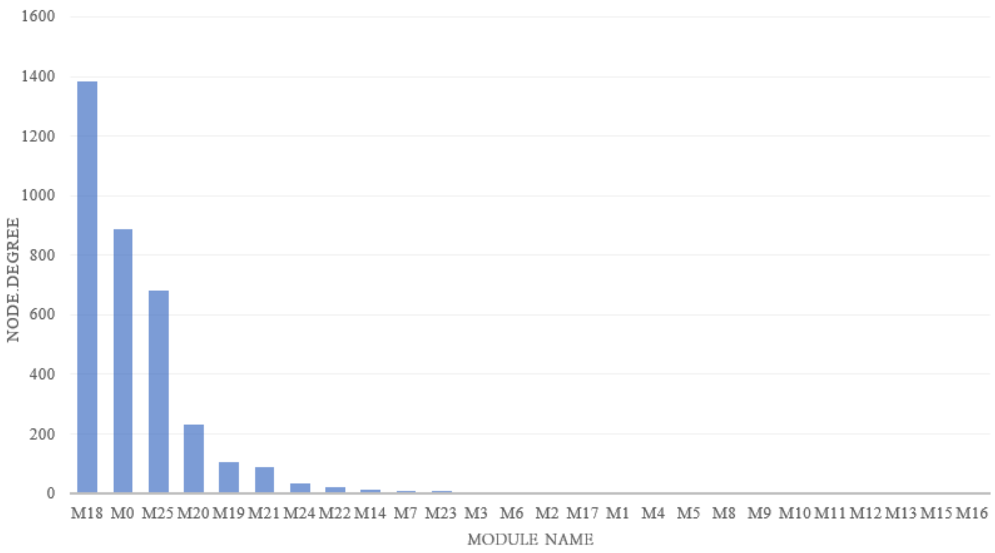

Supplement: Supplementary file 1 [file Data_sheet_1.pdf]
